# The immunoglobulin heavy chain VH6-1 promoter regulates Ig transcription in non-B cells

**DOI:** 10.1186/s12935-014-0114-8

**Published:** 2014-11-26

**Authors:** Lina Wu, Yang Liu, Xiaohui Zhu, Li Zhang, Jinfeng Chen, Hong Zhang, Peng Hao, Shuai Zhang, Jing Huang, Jie Zheng, Yingmei Zhang, Youhui Zhang, Xiaoyan Qiu

**Affiliations:** Key laboratory of Carcinogenesis and Translational Research (Ministry of Education), Central Laboratory, Peking University Cancer Hospital & Institute, Beijing, 100142 China; Peking University Center for Human Disease Genomics, Beijing, 100038 China; Key laboratory of Carcinogenesis and Translational Research (Ministry of Education), Department of Thoracic Surgery II, Peking University Cancer Hospital & Institute, Beijing, 100142 China; Department of Immunology, Cancer Institute, Chinese Academy of Medical Science and Peking Union Medical College, Beijing, 100021 China

**Keywords:** VH6-1, Promoter activity, Oct-1, Transcriptional regulation, Non-B cells

## Abstract

**Background:**

Non-B cell immunoglobulins (Igs) are widely expressed in epithelial cancer cells. The past 20 years of research have demonstrated that non-B cell Igs are associated with cancer cell proliferation, the cellular cytoskeleton and cancer stem cells. In this study we explored the transcriptional mechanism of IgM production in non-B cells.

**Methods:**

The promoter region of a V-segment of the heavy mu chain gene (VH6-1) was cloned from a colon cancer cell line HT-29. Next, the promoter activities in non-B cells and B-cells were detected using the dual-luciferase reporter assay. Then the transcription factor binding to the promoter regions was evaluated by electrophoretic mobility shift assays (EMSAs) and gel supershift experiments.

**Results:**

Our data showed that the sequence 1200 bp upstream of VH6-1 exhibited promoter activity in both B and non-B cells. No new regulatory elements were identified within the region 1200 bp to 300 bp upstream of VH6-1. In addition, Oct-1 was found to bind to the octamer element of the Ig gene promoter in cancer cells, in contrast to B cells, which utilize the transcriptional factor Oct-2.

**Conclusion:**

The regulatory mechanisms among different cell types controlling the production of IgM heavy chains are worth discussing.

## Introduction

Immunoglobulins (Igs) are important immune molecules that are produced when B cells transition into plasma cells. As unique molecules produced by B cells, Igs are also referred to as B cell receptors (BCRs) and play a role in antigen recognition. However, Qiu et al. found that Igs, including IgG, IgA and IgM, are also widely expressed in other types of cells such as normal or cancer cells derived from epithelial tissue, mesenchymal tissue cells and blood myeloid cells and that they are implicated in cell proliferation and carcinogenesis [1,2]. The phenomenon of non-B cells expressing Igs has been confirmed elsewhere [3-7]. Babbage et al. noted the presence of functional transcripts of Ig variable (V), diversity (D) and joining (J) rearrangements in four out of six breast cancer cell lines and sequential cultures, indicating stable expression. These cell lines expressed activation-induced cytidinedeaminase (AID), which is essential for mutational and switch activity [8]. Additionally, using a rat model of breast cancer, Adamovic et al. found that the Ig heavy chain variable region gene is closely associated with breast cancer [9].

Regulation of transcription is thought to involve the interplay between tissue- and developmental-specific transcription factors (TFs), which act upon enhancer and promoter sequences to facilitate the assembly of the transcription machinery at gene promoters. The recombined IgH gene has a relatively simple promoter (referred to as the VH promoter) that is comprised primarily of a conserved TATA box at approximately −30 bp and a highly conserved DNA sequence element (the octamer) at approximately −70 bp relative to the transcription start site [10]. The octamer element is usually located within 100 bp of the transcription initiation site for all VH and Vk promoters [11]. A point mutation in the octamer DNA motif reduced the expression of an Ig transgene by more than 20-fold, as shown in a previous study utilizing transgenic mouse models [12]. POU domain activator proteins have been shown to bind the octamer motif, including both Oct-1 and Oct-2 [13,14]. While Oct-2 is B cell specific and is known to be a major tissue-specific regulator of Ig transcription, Oct-1 is ubiquitously expressed in non-B cells and regulates the expression of housekeeping genes such as histone H2B and snRNA via recognition of the conserved octamer element.

B cell-specific IgH regulation is well characterized, but the regulation of IgH in non-B cells remains unclear. Based on our initial data, which demonstrated that Ig VH genes were frequently expressed in epithelial cancer cells, we undertook a series of studies to explore the mechanisms underlying non-B cell Ig expression. Expression of the VH4-59 segment, a component of IgG heavy chain, was detected in several epithelial cancer cell lines and was found to be driven by Oct-1 [15]. IgM heavy chain expression was present in some primary epithelial cancer cells and epithelial cancer cell lines, and, interestingly, these IgM heavy chains preferentially selected another VH6-1 segment [16]. In this study, we explored the regulatory mechanisms responsible for Ig VH6-1 gene transcription in epithelial cancer cells. We constructed a 5′ upstream 1200-bp fragment of VH6-1 containing the IgH promoter and found that it exhibited promoter activity in all non-B cell lines tested except Jurkat. Unlike the upstream VH4-59 promoter, which contains two novel up-regulatory elements, we detected no novel regulatory element within the region 300 bp to 1200 bp upstream of the VH6-1 promoter in non-B cell cell lines. In addition, we found that Oct-1 but not Oct-2 was a key TF for VH6-1 gene transcription in non-B cells. This observation suggests that the regulatory mechanism of IgG heavy chain is different from that of IgM heavy chain in non-B cells.

## Methods

### Cell lines

The ten cell lines used in this study were supplied by the Peking University Center for Human Disease Genomics. HeLa, HeLa MR, HeLa S3 (all human cervical cancer cell lines), HT-29 (a human colon cancer cell line), MDA-MB-231 (a breast cancer cell line), HEK293 (A human embryonic kidney cell line), HK2 (an immortalized proximal tubule epithelial cell line) and L02 (a human embryonic liver cell line) were cultured in DMEM (Gibco/Invitrogen, Carlsbad, CA, USA) supplemented with 10% fetal bovine serum (FBS) (Hyclone/Thermo Fisher Scientific, Pittsburgh, PA) and 2 mM glutamine. Raji and Daudi (a B lymphocytic leukemia cell line) and Jurkat (T lymphocytic leukemia cell line) cells were cultured in RPMI 1640 medium (Gibco) supplemented as described above. All of the cells were maintained in an incubator with 5% CO_2_ at 37°C.

### Construction of reporter constructs

For the construction of reporter constructs, a fragment containing the human Ig VH6-1 gene core promoter element (including the atypical octamer motif) was amplified from the HT-29 cell line using the following primers: 5′-GGGGTACCCACACAGTGCAGTTTCCACGT-3′ (forward) and 5′-GCGAGATCTCTGGTGACTGCCCTGGTC-3′ (reverse). After sequencing to confirm the fragment, the PCR product was subcloned into the pGL3 vector (Promega, Madison, WI) to create a 1.2-kb pGL3 luciferase reporter plasmid (pGL3-1.2 kb). A 300-bp version of pGL3 (pLG3-300 bp) was generated from the 1.2-kb pGL3 by PCR amplification using the forward primer 5′-GGGGTACCTTTGGTTGATAGGACGCC-3′ and the reverse primer 5′-GCGAGATCTCTGGTGACTGCCCTGGTC-3′. To detect the activity of the octamer element, four base pairs located in the middle of the octamer motif (AGGCAAAT), which is present in the promoter region of the VH6-1 gene, were deleted using the QuikChange site-directed mutagenesis kit (Stratagene/Agilent Technologies, La Jolla, CA,USA). The constructs containing the GCAA deletion were designated as the pGL3-1.2 kb-mt. The sequences of the constructs were confirmed by DNA sequencing.

### Transient transfection

For assays using the pGL3 reporter plasmids, the pGL3 construct was cotransfected with the internal control plasmid pRL-TK (Promega). Firefly and renilla luciferase activities were measured with a dual-luciferase reporter assay kit (Promega). A 20-ms electrical pulse at 120 V was delivered to HeLa, HeLa MR and HeLa S3 cells, while 130 V was delivered to HT-29 cells and 140 V was delivered to Raji and Jurkat cells. After standing at room temperature for 10 min, the transfected cells were allowed to recover at 37°C in DMEM or RPMI-1640 culture medium supplemented with 10% FBS for approximately 30 h in 24-well culture plates. MDA-MB-231, HEK293, L02 and HK2 were transfected with lipofetamine 2000 for 24 h. Luminescence was measured using a Veritasmicroplateluminometer (Turner Biosystem, Sunnyvale, CA, USA). Relative luciferase activity was calculated by measuring the ratio of firefly to renilla luciferase activities.

### RT-PCR

Total RNA was extracted from HT-29, HeLa S3, Jurkat and Daudi cells using TRIzol reagent (Invitrogen, Carlsbad, CA, USA). Reverse transcription (RT) of total RNA from each of these samples was performed using a Superscript II RT kit (Invitrogen) according to the manufacturer’s instructions. The Oct-2 gene was amplified using the forward primer 5′-CCTGCTCAGTTCCTGCTACC-3′ and the reverse primer 5′-GATGCTGGTCCTCTTCTTGC-3′. The PCR reaction for Oct-2 amplification was carried out with one cycle of 94°C for 5 min followed by 30 cycles of 94°C for 30 sec, 54°C for 30 sec and 72°C for 30 sec, finishing with a one-cycle extension step at 72°C for 7 min. PCR products were separated via electrophoresis in a 1.0% agarose gel and visualized by ethidium bromide (EB) staining.

### Electrophoretic mobility shift assays (EMSAs) and gel supershift experiments

EMSAs were performed with Oct proteins. The binding sequence-specific probes 5′-GCAAGTGACGCACACAGGCAAATGCCAGGGTGTGGTTTCC-3′ and 5′-GGAAACCACACCCTGGCATTTGCCTGTGTGCGTCACTTGC-3′ were synthesized with 3′ overhangs, annealed and end-labeled with DIG (second-generation DIG gel shift kit; Roche, Basel, Switzerland). Briefly, after each treatment, cells were harvested and washed twice with cold PBS. The cell pellets were resuspended in 400 μl of cold buffer A (10 mMHepes, 10 mMKCl, 0.1 mM EDTA, 0.1 mM EGTA, 1 mM DTT, 0.5 mM PMSF, pH 7.9). The cells were allowed to swell on ice for 15 min, then 25 μl of 10% NP-40 was added, and the tubes were vortexed vigorously for 10 s. The homogenates were centrifuged at 10,000 g for 30 s. The nuclear pellets were resuspended in 50 μl of ice-cold buffer B (20 mMHepes, 0.4 M NaCl, 1 mM EDTA, 1mM EGTA, 1 mM DTT, 1 mM PMSF, pH 7.9). After vigorously shaking at 4°C for 15 min, the nuclear extracts were centrifuged at 10,000 g for 5 min at 4°C, and the supernatants were collected. The protein concentrations were determined by Bradford analysis. EMSAs were performed using nuclear extracts from HT-29 cells according to the manufacturer’s instructions (Chemiluminescent Nucleic Acid Detection Module, Thermo Fisher/Pierce, Rockford, IL, USA). Supershift assays were performed by preincubating nuclear extracts with 1 μl polyclonal anti-Oct-1 or anti-Oct-2 antibody (Santa Cruz Biotechnology, Santa Cruz, CA, USA) at room temperature for 20 min prior to adding the DNA probes.

### Chromatin immunoprecipitation

In brief, after crosslinking for 30 min with 1% formaldehyde, glycine was added to a final concentration of 125 mM and incubated for 5 min at room temperature. After then incubating at room temperature for 20 min and then at 4°C overnight with gentle agitation, the cells were rinsed twice in PBS, trypsinized, and lysed for 10 min on ice in cell lysis buffer (50 mMHepes-KOH, 140 mMNaCl, 1 mM EDTA, 1% Triton X-100, 10% glycerol, 0.5% NP-40, freshly added protease inhibitors, pH 7.5). After centrifuging at 4,000 rpm at 4°C for 10 min, the nuclei in the pellets were resuspended in nuclear lysis buffer (200 mMNaCl, 1 mM EDTA, 0.5 mM EGTA, 10 mMTris, freshly added protease inhibitors, pH, 8.0) on ice for 10 min. The chromatin was sheared by sonication to an average DNA sequence length of 300–1,000 bp. Each sample was subjected to pre-clearance for 2 h by incubation with protein G-sepharose beads (50% slurry) at 4°C. The pre-cleared samples were incubated with 1.5 mg of anti-Oct-1 at 4°C overnight followed by incubation with 20 ml of pre-blocked protein G beads (50% slurry) at 4°C for 2 h. The precipitates were washed with washing buffer (50 mMHepes, 140 mMNaCl, 1 M EDTA, 1% Triton X-100, 0.1% DOC, freshly added protease inhibitors, pH 7.5) five times and were then resuspended in 100 μl of TE buffer. The combined eluates as well as the input samples (2% of the amount used in the IP procedure) were reverse crosslinked by heating overnight at 65°C in the presence of 200 mMNaCl. After proteinase K digestion, the chromatin fragments were purified, and a 2-μl aliquot was used in each PCR reaction with primer sequences that amplified a 220-bp DNA fragment containing the octamer motif located in the IgH promoter of VH6-1 (forward sequence, 5′-GCTGAAGAACATGGCTCTAGG-3′; reverse sequence, 5′-TGTCTGGAGCTCTGGTGACTG-3′). PCR products were separated by electrophoresis in a 1.0% agarose gel and visualized by EB staining.

### Statistical analysis

Data are expressed as the mean ± standard error (SE) using the GraphPad prism software program (GraphPad Software, La Jolla, CA, USA). All data were obtained from at least duplicate measures for at least three separate and independent experiments.

## Results

### The 1200 bp fragment upstream of VH6-1 exhibits promoter activity in non-B cells

**Figure 1:**
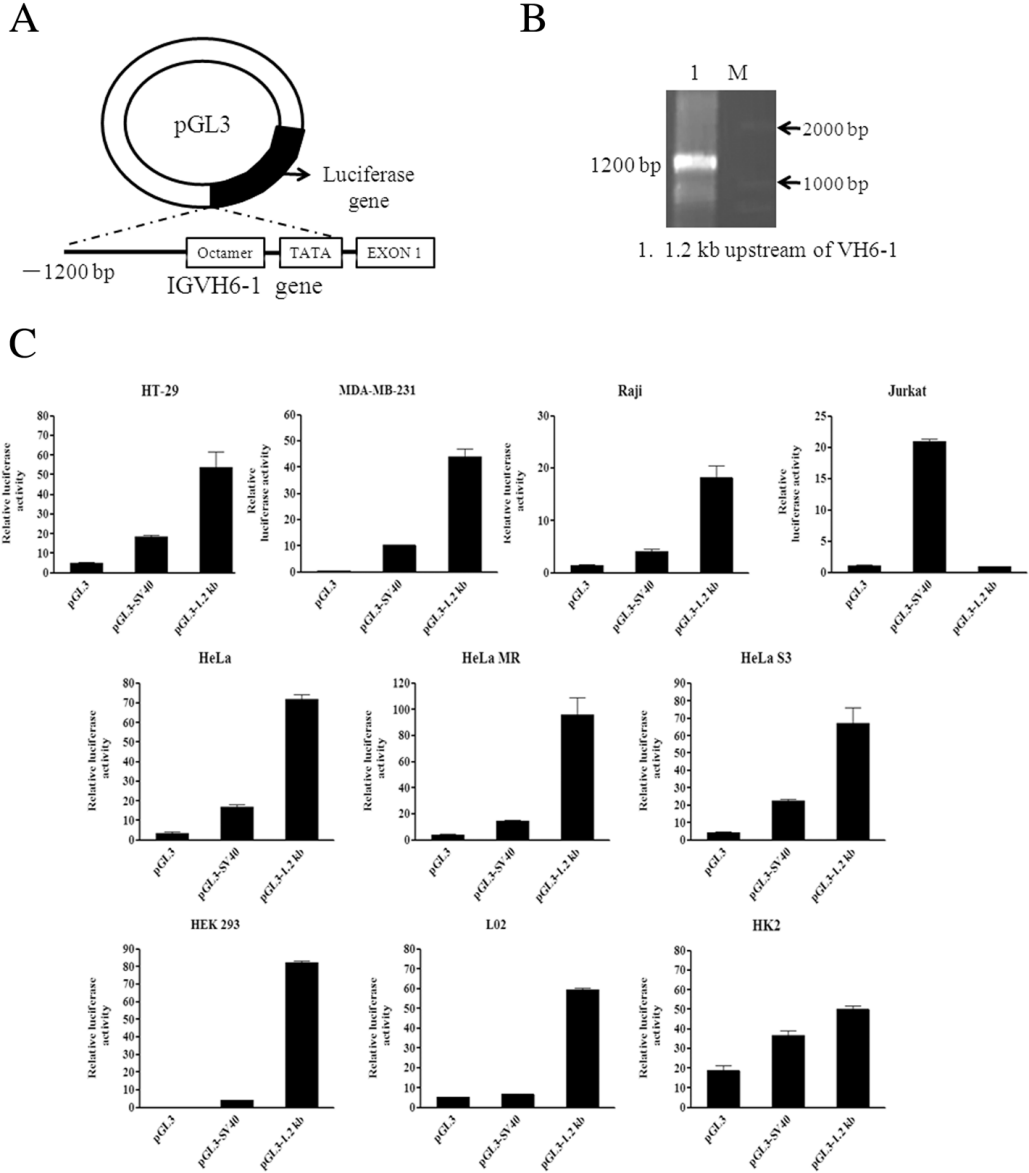
**The 5′-flanking sequence of VH6-1 exhibits promoter activity in non-B cancer cells. (A)** Schematic diagram of 5′-flanking 1.2-kb pGL3 construct. **(B)** The 1.2-kb fragments amplified from upstream of VH6-1 in HT-29 cells by PCR. **(C)** The 1.2-kb pGL3 construct was transfected into HeLa, HeLa MR, HeLa S3, HT-29, MDA-MB-231, HEK 293,L02, HK2, Raji or Jurkat cells. Luciferase activity was measured using a dual-luciferase reporter system. The results are representative of three independent experiments after normalization to renilla luciferase activity (internal controls). Each bar represents mean ± SD.

The Ig VH core promoter is primarily composed of a conserved TATA box and the octamer element that is located within 100 bp of the transcriptional start site. We first amplified the upstream 1.2-kb DNA fragment of VH6-1 from HT-29 (a colon cancer cell line) by PCR and cloned this fragment into a pGL3 luciferase reporter vector named pGL3-1.2 kb (Figure 1A&B). To examine the promoter activity of pGL3-1.2 kb, dual-luciferase reporter assays were performed in ten cell lines, including HT-29, three cervical cancer cell lines HeLa, HeLa MR and HeLa S3,a breast cancer cell line MDA-MB-231, three immortalized diploid cell lines HEK293, L02 and HK2, Raji cells as a positive control and Jurkat cells as a negative control. The 5′ flanking sequence of VH6-1 demonstrated higher promoter activity in nine cell lines than the pGL3 control and even exhibited higher activity than the Simian Virus 40 (SV40) promoter, which demonstrated high activity in mammalian cell lines (Figure 1C). As expected, the 1.2-kb construct exhibited no activity in Jurkat cells.

### The octamer element is important but not essential in non-B cells

**Figure 2:**
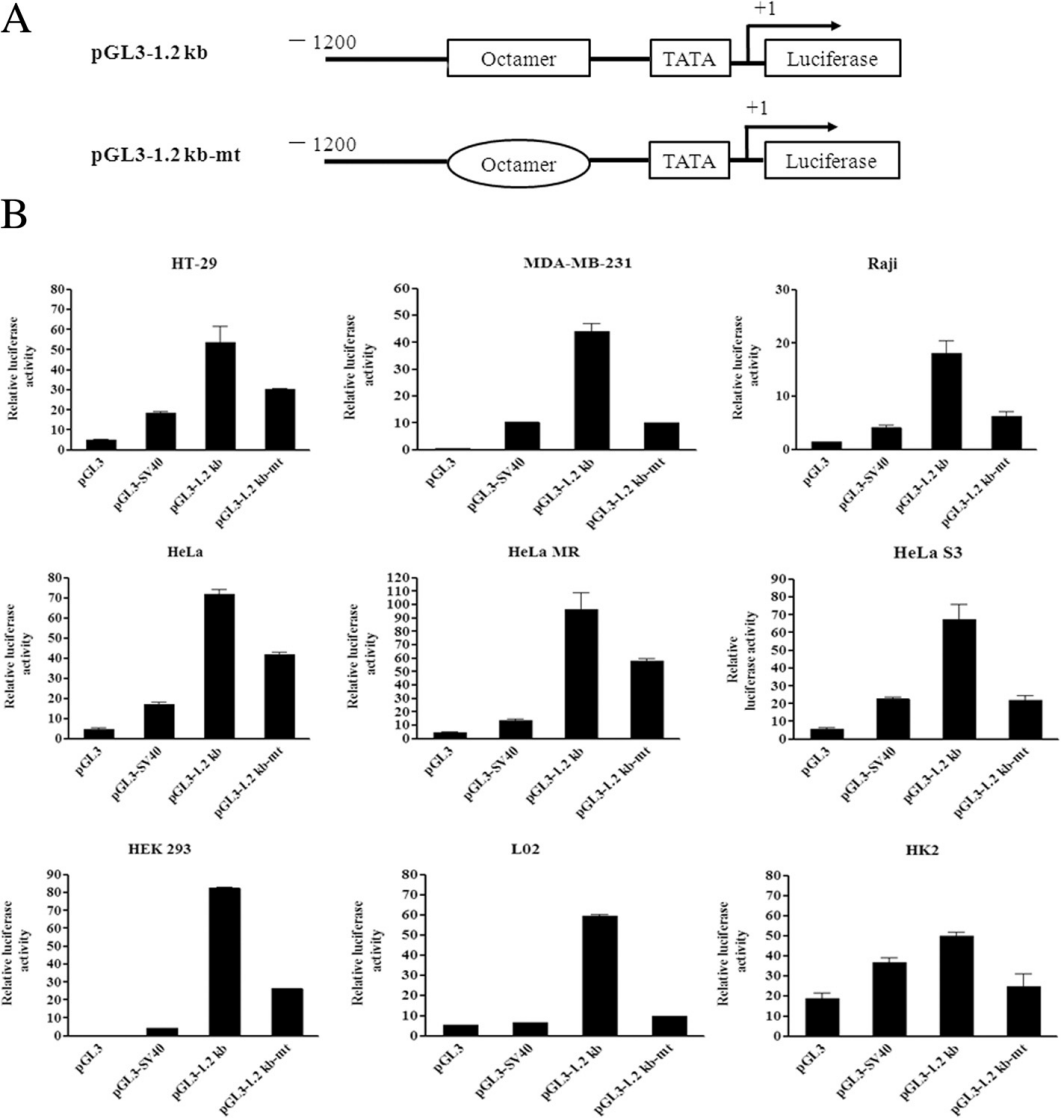
**The octamer element is important but not essential for non-B cell-derived Ig gene transcription. (A)** Schematic diagram of mutated 5′ deletion truncations of the 1.2-kb pGL3 construct with a 4-bp deletion in the octamer motif (AGGCAAAT). **(B)** The intact and mutated 5′ deletion truncations of the 1.2-kb pGL3 construct were transfected into HeLa, HeLa MR, HeLa S3, HT-29, MDA-MB-231, HEK 293, L02, HK2, and Raji cells. Luciferase activity was measured using a dual-luciferase reporter system. The results are representative of three independent experiments after normalization to renilla luciferase activity (internal controls). Each bar represents mean ± SD.

There is an atypical octamer sequence in VH6-1 (AGGCAAAT) that deviates from the typical sequence (ATGCAAAT). To ascertain the function of the octamer elements located in the Ig VH6-1 promoter, a mutated plasmid (pGL3-1.2 kb-mt) was constructed by deleting the middle four base pairs of the octamer element (Figure 2A). Unexpectedly, the deletion construct still exhibited promoter activity, although the activity was lower than for the construct that included the complete promoter construct pGL3-1.2 kb in all five cancer cells, three immortalized diploid cells and Raji cells (Figure 2B). These results indicated that the octamer element of VH6-1 in non-B cells is important but not essential.

### The sequence 1200 bp to 300 bp upstream of VH6-1 contains no regulatory elements

**Figure 3:**
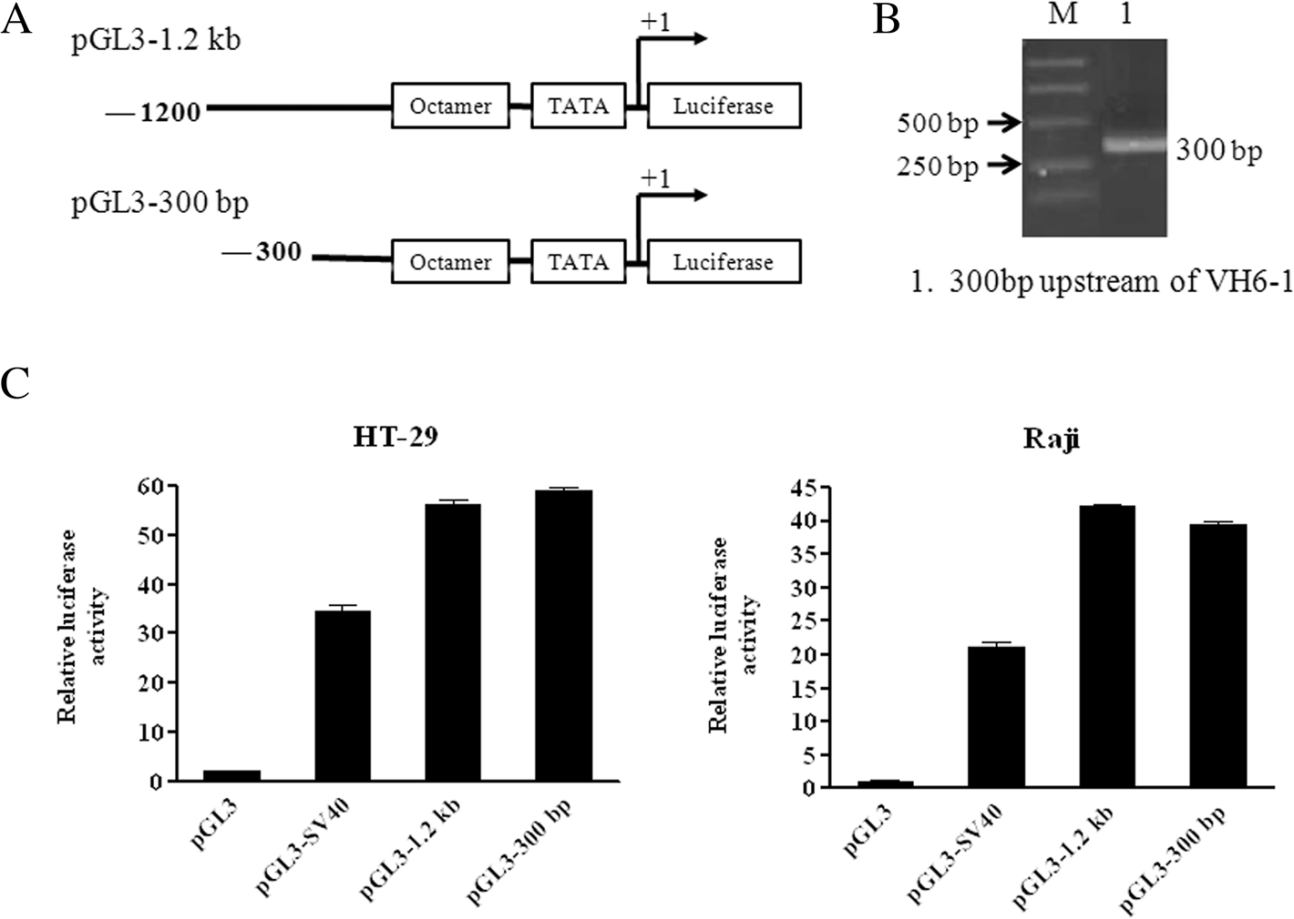
**The sequence 1200 bp to 300 bp upstream of VH6-1 contains no regulatory elements. (A)** Schematic diagram of 5′-flanking 300-bp pGL3 construct. **(B)** The 300-bp deletion fragment amplified from the 1.2-kb fragment containing the VH6-1 promoter by PCR. **(C)** The 300-bp deletion truncations of the 1.2-kb pGL3 construct exhibited similar promoter activity to the 1.2-kb pGL3 construct in HT-29 and Raji cells.

It was previously shown that the region 1200 bp to 300 bp upstream of VH4-59 contains two regulatory elements [15]. To determine whether regulatory elements exist in the sequence upstream of the VH6-1 promoter, the 1200-bp region containing the VH6-1 promoter was deleted via PCR (Figure 3A&B). The promoter activity of the deletion construct (pGL3-300 bp) was tested using a dual-luciferase reporter assay. Strong promoter activity was observed for both the 1200bb and 300-bp constructs in HT-29 and Raji cells, and the promoter activities of both were higher than for the SV40 promoter (Figure 3C). It is likely that there are no regulatory elements upstream (between 1200 bp and 300 bp) of the promoter of VH6-1 in cancer cells. This suggests that the mechanisms regulating the expression of VH6-1 and VH4-59 are different.

### Oct-1, but not Oct-2, regulates VH6-1 promoter activity

**Figure 4:**
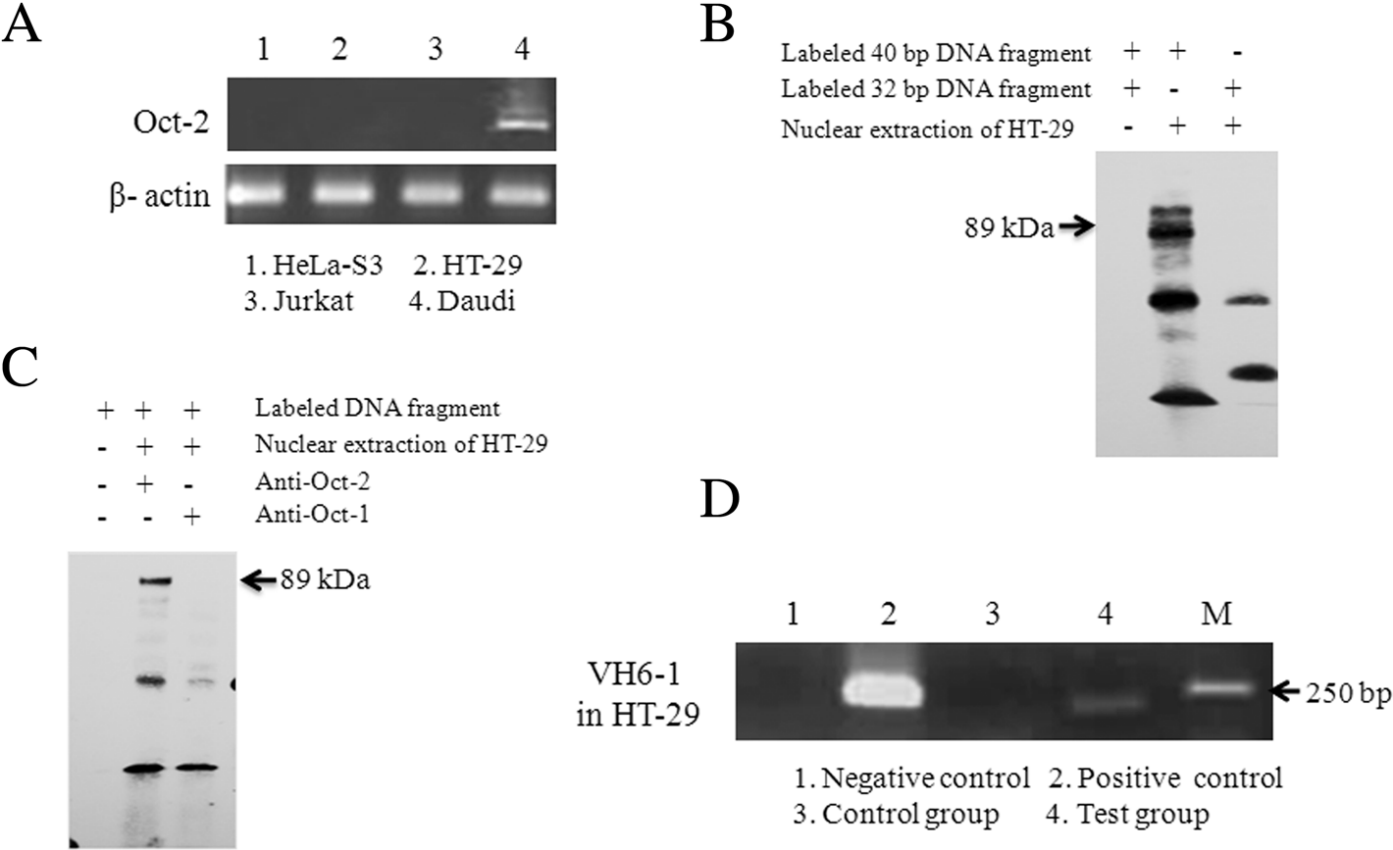
**Oct-1 but not Oct-2 binds to the octamer element. (A)** Oct-2 was not detected in epithelial cancer cells by RT-PCR. **(B)** The EMSA assay for octamer motif binding factors located in the promoter region of VH6-1 in HT-29 cells. The 40-bp DNA fragment was derived from upstream of the VH6-1 gene and contains the octamer motif, while the 32-bp DNA fragment was derived from the 40-bp DNA fragment with an 8-bp deletion in the octamer motif. **(C)** The super-shift assay for octamer motif binding factors with the addition of an anti-Oct-1 or anti-Oct-2 antibody in the binding reaction system. The results are representative of three independent experiments. EMSA, electrophoretic mobility shift assay. **(D)** The Oct-1 binding DNA fragment of the VH6-1 promoter was amplified via Chip-related PCR. Negative control: no template in the PCR reaction system; positive control: the sonicated chromatin fragments of the cells were used as the PCR template; control group: no antibody added to the IP system; test group: dilutions of IP were used as templates for PCR to amplify the Oct-1 binding DNA sequence. The results are representative of three independent experiments.

It is known that Oct-2 binds to the octamer element in mature B lymphocytes to initiate the expression of Ig. The Oct-2 transcript was not detected by RT-PCR in HeLa S3, HT-29 and Jurkat cells, compared with Daudi cells as a positive control (Figure 4A). Next, EMSAs were performed to evaluate the binding of TFs to the octamer element of the Ig VH6-1 gene in epithelial cancer cells. Nuclear protein fractions from HT-29 cells interacted with octamer elements of the VH6-1 upstream region, appearing in a non-denaturing electrophoresis hysteresis band. Compared with the 40-bp double-stranded oligonucleotides containing the octamer motif, the hysteresis band disappeared for the 32-bp oligonucleotides that did not contain an octamer motif, indicating that this interaction was specific (Figure 4B). The anti-Oct-1 antibody strongly super-shifted the binding complex of the HT-29 nuclear proteins, which indicated that no hysteresis band was detected because the DNA binding to the antibody-protein complex did not exit through holes (Figure 4C). These results also indicated that Oct-1 interacts with the octamer element to promote Ig VH6-1 expression in HT-29 cells.

To confirm that the endogenous Ig VH6-1 promoter was bound by endogenous Oct-1, ChIP assays were performed with Oct-1-specific antibodies. The VH6-1 promoter could be amplified from immunoprecipitated complexes obtained with anti-Oct-1 antibodies but not from control complexes in the absence of an antibody (Figure 4D). Oct-1 therefore bound the endogenous octamer element for Ig VH6-1 transcription in HT-29 cells.

## Discussion

In 1996, Qiu et al. found that IgG was expressed in breast cancer cells, and the past 20 years of research have shown that IgG is widely expressed in almost all epithelial carcinomas and epithelial cancer cell lines, as well as in some normal epithelial cells, neurons and germ cells from patients without cancer and in healthy mice [2,17,18]. Moreover, cancer-derived Ig heavy chains exhibited differential repertoires for the gamma chain and the mu chain in the same cells. The Ig VH5-51, VH4-59 and VH3-30 genes were frequently used by the gamma chain, and the VH6-1 gene was frequently used by the mu chain in epithelial cancer cells [16]. The classical theory that Igs are the unique products of B cells has been challenged by these observations. However, the detailed mechanisms of non-B cell Ig production remain unclear.

In B lymphocytes, recombinatorial IgH transcriptional regulation primarily involves a looping mechanism for enhancer-promoter communication. It was thought that transcription of the Ig gene was silenced in non-B cells. In 1994, Sun et al. discovered that the VH6-1 promoter was very active in HeLa cells. Because this was inconsistent with classical immunoglobulin theory, they speculated that the VH6-1 promoter did not possess tissue specificity and that opening of the chromatin in this area upstream of the diversity region may specially require the product of the VH6 transcript [18]. However, our data, and those of other investigators, showed that functional transcripts of Ig VDJ rearrangements were present in both primary cancer cells and cancer cell lines such as HeLa [16]. In our previous study, by immunohistochemistry, we detected IgM expression in many epithelial cancer tissues and their corresponding normal tissues, and found that IgM was expressed in epithelial cancer cells with a high frequency than that in normal epithelial cells although IgM was expressed in both epithelial normal or cancer cells [19]. In our study, the VH6-1 promoter derived from HT-29 cells was very active in several epithelial cancer cell lines and immortalized diploid cells, indicating that Ig VH genes were located in the open chromatin DNA region and that non-B cells can also drive Ig gene transcription. Maybe the promoter activity of VH6-1 can be revealed in some normal cells in the body with a lower level than that in their corresponding cancer cells.

In addition to the core promoter elements that include the consensus TATA box and the Inr element, the hallmark of all VH promoters is the presence of an octamer element. The best-characterized IgH enhancers are intronic (Eμ) enhancers located between the 3′-most joining (J) and constant μ (Cμ) regions and 3′ enhancers (located downstream of Cα, here referred to as the 3′-RR) that are spread over a 30-kb region. The remarkable conservation of the octamer element in both Ig light and heavy chain gene promoters, together with its appearance in both Eμ and 3′-RR, has led to the assumption that transcription factor interactions with this element are crucial for regulation of Ig genes. B cell-specific TF binding to the octamer element occurs with Oct-2. With EMSA and ChIP assays, we found that Oct-1, but not Oct-2, bound to the atypical octamer element (5′-AGGCAAAT-3′) of VH6-1 in cancer cell lines. However, we confirmed that although the octamer element was very important for Ig VH6-1 gene transcription in HT-29 cells, high promoter activity was also present when the octamer element was deleted. This result suggests that, unlike previous assumptions, there are other unidentified regulatory elements within the upstream 300-bp region in both B cells and non-B cells. In previous studies, we found two positive regulatory elements in the upstream promoter region of VH4-59 in epithelial cancer cell lines but not in B cells that were used as positive controls [15]. Utilizing a series of 5′ flanking sequences of the VH6-1 promoter, we observed that unlike VH4-59, no other activation elements are located within 1200 bp to 300 bp upstream of the VH6-1 promoter, which is similar to that found in B cells. Therefore, it is worth investigating the functions and mechanisms of action of additional TFs involved in Ig gene transcriptional regulation in non-B cells.
